# P01 Assessment of risk factors associated with MDR organism infections among patients admitted in a tertiary hospital—a retrospective study

**DOI:** 10.1093/jacamr/dlae136.005

**Published:** 2024-08-23

**Authors:** Alaa Alsehemi, Emad Alharbi, Buthainah Alammash, Alaa Alrais, Yaser M Alahmadi

**Affiliations:** Department of Pharmaceutical Care, Prince Sultan Armed Forces Hospital, Madinah, Saudi Arabia; Department of Pharmaceutical Care, King Fahad Hospital, Madinah, Saudi Arabia; Department of Pharmaceutical Care, King Fahad Hospital, Madinah, Saudi Arabia; Department of Pharmaceutical Care, King Fahad Hospital, Madinah, Saudi Arabia; Department of Pharmacy Practice, College of Pharmacy, Taibah University, Madinah, Saudi Arabia

## Abstract

**Background:**

The misuse of antibiotics has contributed to the worldwide rise in bacterial resistance seen in the medical community. The prevalence of MDR organisms (MDROs) has been growing rapidly in recent years in Saudi Arabia, prompting us to conduct this investigation.

**Objectives:**

This retrospective, observational, case-control study aimed to determine and identify the common risk factors of MDROs among admitted patients to develop a predictive model of infection by MDROs that may help in selecting the appropriate initial antibiotic treatment.

**Methods:**

In total 270 patients were included; they were all adults ≥18 year old with positive bacterial culture. A total of 136 out of 270 individuals tested positive for MDROs, and CRE (24.3%) and XDR (20.6%) strains were determined to be the most prevalent types. Patients who were male and ≤65 years old made up the majority of the population. Among comorbidities, stroke and hemiplegia were more prevalent with MDROs and prior IV antibiotic use at 3 months was greater. Furthermore, medical devices were significantly more associated with MDRO cases. In addition, the usage of acid suppression medications, corticosteroids and a history of prior surgery was more prevalent in MDRO cases. *Klebsiella pneumoniae* was the most common pathogen (30.9%), followed by *Acinetobacter baumannii* (20.6%) and *Staphylococcus aureus* (19.1%) in the case group.

**Conclusions:**

These findings show that stroke, hemiplegia, use of medical devices, previous use of corticosteroids, acid suppressive medications and previous IV antibiotics were associated with high risk of MDRO infections. Knowing the risk factors for MDROs and giving the right antibiotic treatment immediately may help reduce the number of cases and deaths from these infections.

